# Changes in disability in people with multiple sclerosis: a 10-year prospective study

**DOI:** 10.1007/s00415-017-8676-8

**Published:** 2017-11-20

**Authors:** David Conradsson, Charlotte Ytterberg, Lena von Koch, Sverker Johansson

**Affiliations:** 1 Division of Physiotherapy, Department of Neurobiology, Care Sciences and Society, Karolinska Institutet, 23100, Huddinge, 141 83 Stockholm, Sweden; 2 Allied Health Professionals Function, Function Area Occupational Therapy and Physiotherapy, Karolinska University Hospital, Stockholm, Sweden; 3 Division of Occupational Therapy, Department of Neurobiology, Care Sciences and Society, Karolinska Institutet, Stockholm, Sweden; 4 Department of Neurology, Karolinska University Hospital, Stockholm, Sweden

**Keywords:** Disease progression, Epidemiology, Functioning, Longitudinal study, Mobility, Participation

## Abstract

**Background:**

Little is known about the long-term course of disability in relation with disease severity in people with multiple sclerosis (PwMS).

**Objective:**

To explore changes in a broad spectrum of disability over 10 years in relation with disease severity in PwMS.

**Methods:**

We conducted a longitudinal study of 155 PwMS who attended the MS Centre at Karolinska University Hospital, Stockholm. Disease severity was determined by the use of the Expanded Disability Status Scale (EDSS) and classified as mild MS (EDSS score 0–3.5) or moderate/severe MS (EDSS score 4–9.5). Ten-year changes in perceived physical and psychological impacts of MS, walking, cognition, manual dexterity, participation in social/lifestyle activities, and signs of depression were compared between PwMS with mild and moderate/severe MS at baseline.

**Results:**

Although walking, manual dexterity, and cognition declined in both groups, only the moderate/severe group demonstrated that long-term increased physical impact of MS, increased wheel-chair dependency, and reduced participation in social/lifestyle activities. Perceived psychological impact of MS declined in both groups, while signs of depression were experienced by fewer in the mild group and remained unaltered in the moderate/severe group.

**Conclusion:**

We found a more pronounced increase in disability across 10 years in individuals with moderate/severe MS compared to mild MS. These findings accentuate the importance of developing a variety of interventions that can be applied across the spectrum of disease severity.

## Introduction

The progressive disability course of Multiple Sclerosis (MS) is leading to significant individual and societal burdens [1]. As MS typically is diagnosed between the ages of 20 and 40, most people with MS (PwMS) will likely experience disability across different areas of functioning for decades [2]. Therefore, understanding the course across a wide spectrum of disability is required to prioritize and optimize health services for PwMS.

In line with the progressiveness of MS, the previous longitudinal studies have demonstrated increased EDSS scores over time [3–5] and a decline in the physical aspects of quality of life [4, 6, 7]. In contrast, psychological domains of quality of life have been shown to remain stable [7, 8] or even improve [6]. Similarly, although fatigue and signs of depression are common among PwMS, most studies have found unchanged levels over time [7, 9, 10]. Notably, a majority of these studies included a follow-up period of 4–6 years [4, 6, 8, 10], whereas few studies have monitored long-term disability for a decade or more.

Although the aforementioned studies have provided significant information about the course of disability for PwMS, there are important limitations to the existing body of research. First, few studies have addressed the long-term course of disability in different groups of disease severity. In a study by Wynia et al. [8], the authors compared changes in self-reported disability across 5 years between PwMS with different severity levels of MS. While this study found a more prominent increase in disability for individuals with mild MS, a higher mortality rate was observed in the severe disease group [8]. These findings need to be confirmed and self-reported disability complemented with performance-based tests of functioning. Second, cross-sectional studies have demonstrated that disability in walking, manual dexterity, and cognition is associated with increased activity limitations [11, 12], or a decreased quality of life in PwMS [2]. However, few long-term studies have conducted a detailed assessment to evaluate the progression of disability in relation with activities of daily living (ADL) and participation. Finally, although immunomodulatory treatment has shown promising impact on the course of the disease severity in PwMS [13], the previous long-term studies have evaluated cohorts, where a minority have received immunomodulatory treatment (15–40%) [3, 5, 9].

In this explorative study, we aimed to investigate changes in a broad spectrum of disability over 10 years in a cohort of PwMS who, to a large extent, received immunomodulatory treatment. More specifically, we aimed to compare changes concerning disease severity, perceived physical and psychological impacts of MS, walking ability, cognition, manual dexterity, fatigue, signs of depression, ADL, and participation in social/lifestyle activities between individuals with mild MS and with moderate/severe MS at baseline.

## Materials and methods

### Study design and participants

This study was a 10-year prospective study in PwMS, and details of the baseline and short-term follow-ups of this cohort have been published elsewhere [2, 14]. Briefly, during the period between February 2002 and June 2002, consecutive PwMS who were scheduled for an outpatient appointment with a neurologist at the MS Centre of Karolinska University Hospital, Stockholm, Sweden were considered for enrolment in this study. Of the 255 eligible PwMS diagnosed according to Poser criteria [15], 219 agreed to participate. Participants were re-assessed in 2004 and 2012 for a 2- and 10-year follow-up. All participants were contacted and received information about the aim and procedures of the present study, and thereafter, written informed consent was obtained. This study was approved by the regional board of ethics in Stockholm, registration numbers 449/01 and 2011/2068-31/5, and was conducted in accordance with the ethical standards laid down in the 1964 Declaration of Helsinki and its later amendments.

### Data collection

Data collection comprised standardized face-to-face interviews, self-reported questionnaires, and performance-based tests [2]. The 10-year follow-up occurred at the MS Centre or in the participant’s home. Demographic data (age and sex), disease duration (years), symptomatic pharmacological treatment, work status, living situation, and use of mobility devices were collected using standardized questions administered as an interview and from medical records.

The course of MS was classified as relapsing–remitting or progressive MS. Disease severity was determined by the use of the Expanded Disability Status Scale (EDSS) [16] and classified as mild MS (EDSS score 0–3.5) or moderate/severe MS (EDSS score 4–9.5). The mean difference of the EDSS score between baseline and the 10-year follow-up was used to assess change in disease severity. We also monitored two milestones of MS disability: dependency on a walking device (EDSS score ≥ 6) or on a wheelchair (EDSS score ≥ 7).

The Multiple Sclerosis Impact Scale (MSIS-29) was used for assessment of the perceived physical and psychological impacts of MS from the perspective of the PwMS [17, 18]. The sum score of MSIS-29 physical and psychological subscales was analyzed.

Walking ability and manual dexterity were assessed with the Timed 25-Foot Walk [19] and the Nine-Hole Peg Test (NHPT) [20], respectively. For both tests, we calculated an average of two trials with outcomes including maximum speed (m/s) for walking ability and number of pegs per second of the dominant hand for manual dexterity. The Symbol Digit Modalities Test (SDMT) was used to assess cognitive processing by means of the capacity to direct attention quickly and accurately [21]. SDMT was primarily administered in written format, but for PwMS with severe upper extremity limitations, we administered the test orally. The Fatigue Severity Scale was used to assess the severity and impact of fatigue on daily functioning and a mean score of its nine items was analyzed [22]. Mood was assessed with the Beck Depression Inventory (BDI) [23, 24] and a score ≥ 13 was the cutoff for signs of depression [25].

Participation in social/lifestyle activities was assessed with the Frenchay Activities Index covering domestic chores and outdoor, work, and leisure activities that require initiative on the part of the individual [26]. The sum score of the 15 items included was used in the analyses. The Katz ADL Index Extended was used to assess independence in personal ADL (bathing, dressing, toileting, transferring, continence, and feeding) and instrumental ADL (using public transportation, shopping, food preparation, and housekeeping) [27, 28]. Participants who reported dependency in one or more items were classified as dependent in personal or in instrumental ADL.

### Analysis

Statistical analyses were carried out using SAS^®^ (System 9.4, SAS Institute Inc., Cary, NC, USA software) and IBM SPSS, version 23.0 (SPSS Inc., Chicago, Illinois, USA). Descriptive statistics, mean (standard deviation), and numbers (percentages) were used to present demographics (age, sex, living situation, and work status) and MS-related variables (disease duration, EDSS score, disease course, immunomodulatory treatment, and use of a mobility device) at baseline. The Student’s *t* test, Mann–Whitney *U* test, *χ* ^2^, and the Fisher’s exact test were used to compare changes in disease severity, to test for baseline differences between the groups with mild and moderate/severe MS and to compare those who completed the 10-year follow-up with those who dropped out. For disability outcomes, the percentage of missing data due to participants’ inability to complete the protocol was overall small across the study period. Hence, we analyzed all cases and did not exclude participants with missing data at one test occasion. For dichotomized disability outcomes (use of mobility devices, dependency in ADL, and signs of depression), changes in proportions were analyzed with the McNemar test. For continuous disability outcomes, a mixed-model analysis was used to analyze differences in changes between the groups (mild MS vs. moderate/severe MS) and time (baseline, 2- and 10-year follow-up) on physical and psychological impacts of MS, walking ability, cognitive processing, manual dexterity, fatigue, and participation in social/lifestyle activities. In case of a significant interaction effect, independent and paired *t* tests were performed as post hoc tests to assess between and within group differences. The significance level was set at *P* ≤ 0.05.

## Results

### Participants’ baseline characteristics

**Fig. 1:**
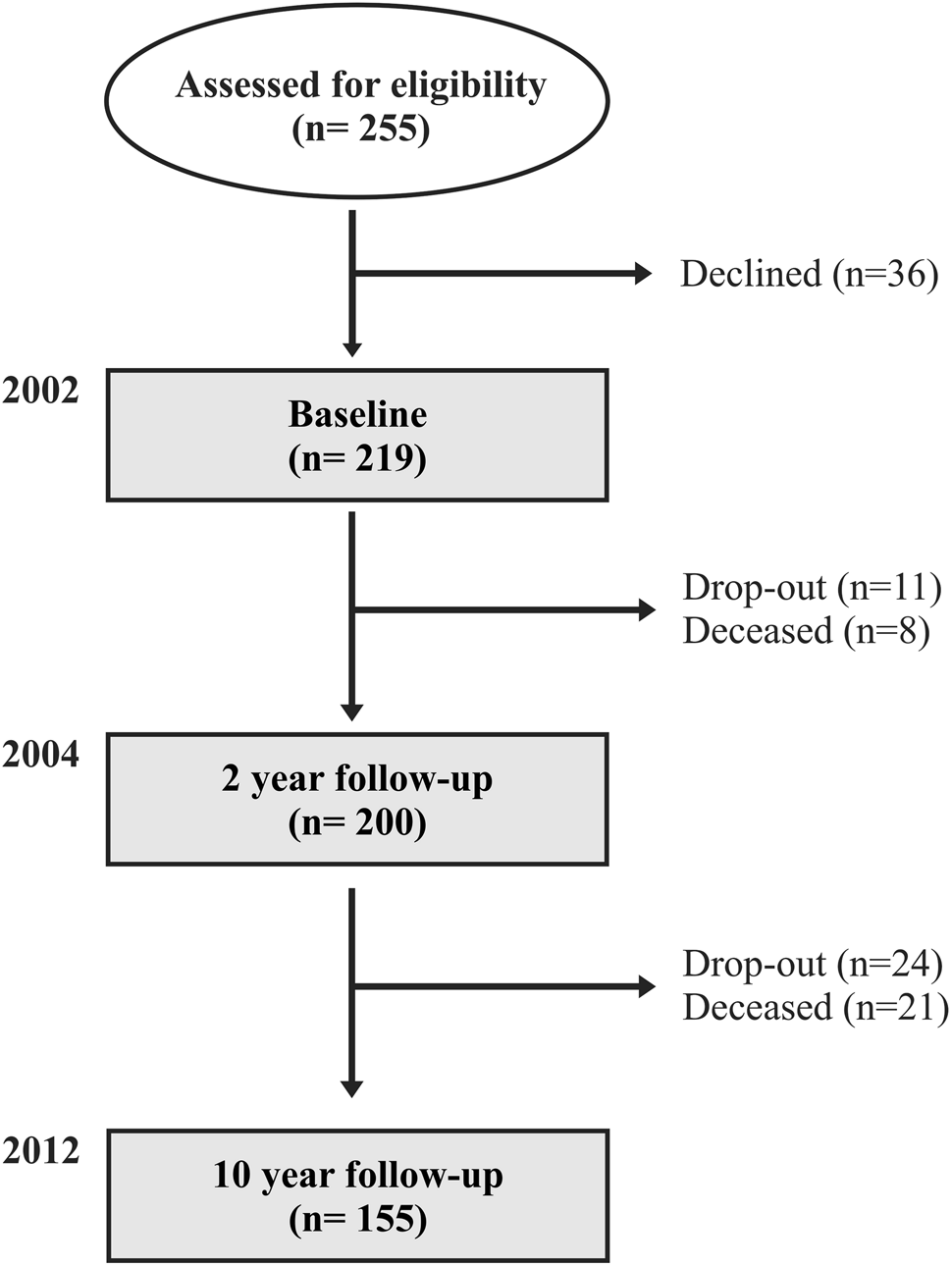
Study flowchart

As illustrated in Fig. 1, of the 219 PwMS enrolled in the study in 2002, 155 participated in the 2- and 10-year follow-up. At baseline, 101 (65%) of the participants were classified as having mild MS and 54 (35%) had moderate/severe MS (see Table 1). The group with moderate/severe MS was 7.6-year older and demonstrated 6.5-year longer disease duration than those in the group with mild MS (*P* < 0.001). Compared to the mild group, a larger proportion of the moderate/severe group received immunomodulatory treatment (94 vs. 81%, *P* = 0.029) at baseline had progressive MS (81 vs. 12%, *P* < 0.001), used a mobility device (walking device: 35 vs. 2%, wheelchair: 24 vs. 0%, *P* < 0.001), and were not working (63 vs. 26%, *P* < 0.001).

**Table 1:** Baseline data for participants with mild, moderate/severe MS completing the study and those lost to the 10-year follow-up

	Mild MS (*n* = 101)	Moderate/severe MS (*n* = 54)	All (*n* = 155)	Lost to follow-up (*n* = 64)
Age (years), mean (SD)*^†^	42.8 (11.5)	50.4 (9.8)	45.4 (11.5)	50.0 (14.0)
Female sex (%)	72 (71)	32 (59)	104 (67)	45 (70)
Living alone (%)^†^	26 (26)	14 (26)	40 (26)	27 (42)
MS duration (years), mean (SD)*^†^	10.8 (8.9)	17.3 (10.2)	13.0 (9.9)	16.5 (11.3)
EDSS score, mean (SD)*^†^	1.7 (0.8)	5.9 (1.5)	3.2 (2.3)	4.6 (2.8)
Immunomodulatory treatment (%)	82 (81)	51 (94)	133 (86)	49 (77)
Disease course*^†^				
Relapsing–remitting (%)	89 (88)	10 (19)	99 (64)	27 (42)
Progressive (%)	12 (12)	44 (81)	56 (36)	37 (58)
Mobility device*^†^				
No device (%)	99 (98)	22 (41)	121 (78)	34 (53)
Walking device (%)	2 (2)	19 (35)	21 (14)	10 (16)
Wheelchair (%)	0 (0)	13 (24)	13 (8)	20 (31)
Work status*^†^				
Working (%)	75 (74)	20 (37)	95 (61)	21 (33)
Not working (%)	26 (26)	34 (63)	60 (39)	43 (67)

*EDSS* Expanded Disability Status Scale, *MS* Multiple Sclerosis

*Significant differences between the group with mild and moderate/severe MS

^†^Significant differences (< 0.05) between those completing the study and those dropping out of the study

### Participants lost to follow-up

Those lost to follow-up were 4.6-year older at baseline and had 3.5-year longer disease duration and 1.4 points higher EDSS scores compared to those completing the study (*P* < 0.037, see Table 1). A larger proportion of those not participating also lived alone (42 vs. 26%, *P* = 0.017) had progressive MS (58 vs. 36%, *P* = 0.003), were dependent on a wheelchair (31 vs. 8%, *P* < 0.001), and were not working (67 vs. 39%, *P* < 0.001).

### Changes in disease severity and disability profiles

The mean change in EDSS scores between baseline and the 10-year follow-up was similar for participants with mild MS (diff: 1.5, 95% CI 1.2–1.8) and moderate/severe MS (diff: 1.3, 95% CI 1.0–1.5, *P* = 0.359). The proportion using symptomatic pharmacological treatment in the cohort at baseline and at 10-year follow-up are presented in Table 2. While the moderate/severe group increased their dependency on walking devices and wheelchairs across the study period (*P* < 0.006), the mild group only increased their dependency on walking devices (*P* < 0.001, Table 3). At the 10-year follow-up, 21 and 3% in the mild group and 94 and 54% in the moderate/severe group revealed dependency on walking devices and wheelchairs, respectively (Table 3).

**Table 2:** Symptomatic pharmacological treatment at baseline and at 10-year follow-up

	Baseline, *n* = 155 (%)	10-year follow-up, *n* = 155 (%)
Pain/flu like symptoms	91 (59)	53 (34)
Spasticity	24 (16)	27 (17)
Depressive symptoms	37 (24)	33 (21)
Urogenital problems	32 (21)	39 (25)
Fatigue	19 (12)	21 (14)
Insomnia	24 (16)	24 (16)

**Table 3:** Proportions (percentages) in the mild and moderate/severe MS groups demonstrating dependency on walking device, wheelchair, activities of daily living, and signs of depression at baseline, 2 and 10-year follow-up

	Mild MS (*n* = 101)			Moderate/severe MS (*n* = 54)		
	Baseline	2 year	10 year	Baseline	2 year	10 year
Walking device	0 (0)	3 (3)	21 (21)†	27 (50)	37 (69)*	51 (94)^†^
Wheelchair	0 (0)	0 (0)	3 (3)	20 (37)	20 (37)	29 (54)^†^
Personal ADL	3 (3)	5 (5)	24 (24)†	20 (36)	23 (42)	40 (73)^†^
Instrumental ADL	14 (14)	16 (16)	41 (41)†	40 (73)	48 (87)*	52 (95)
Depressive symptoms	16 (16)	23 (23)	12 (12)†	16 (29)	12 (22)	14 (25)

*ADL* Activities of Daily Living, *MS* Multiple Sclerosis

*Significant difference (< 0.05) between baseline and the 2-year follow-up

^†^Significant difference (< 0.05) between the 2 and 10-year follow-up

### Changes in physical and psychological impacts of MS

**Fig. 2:**
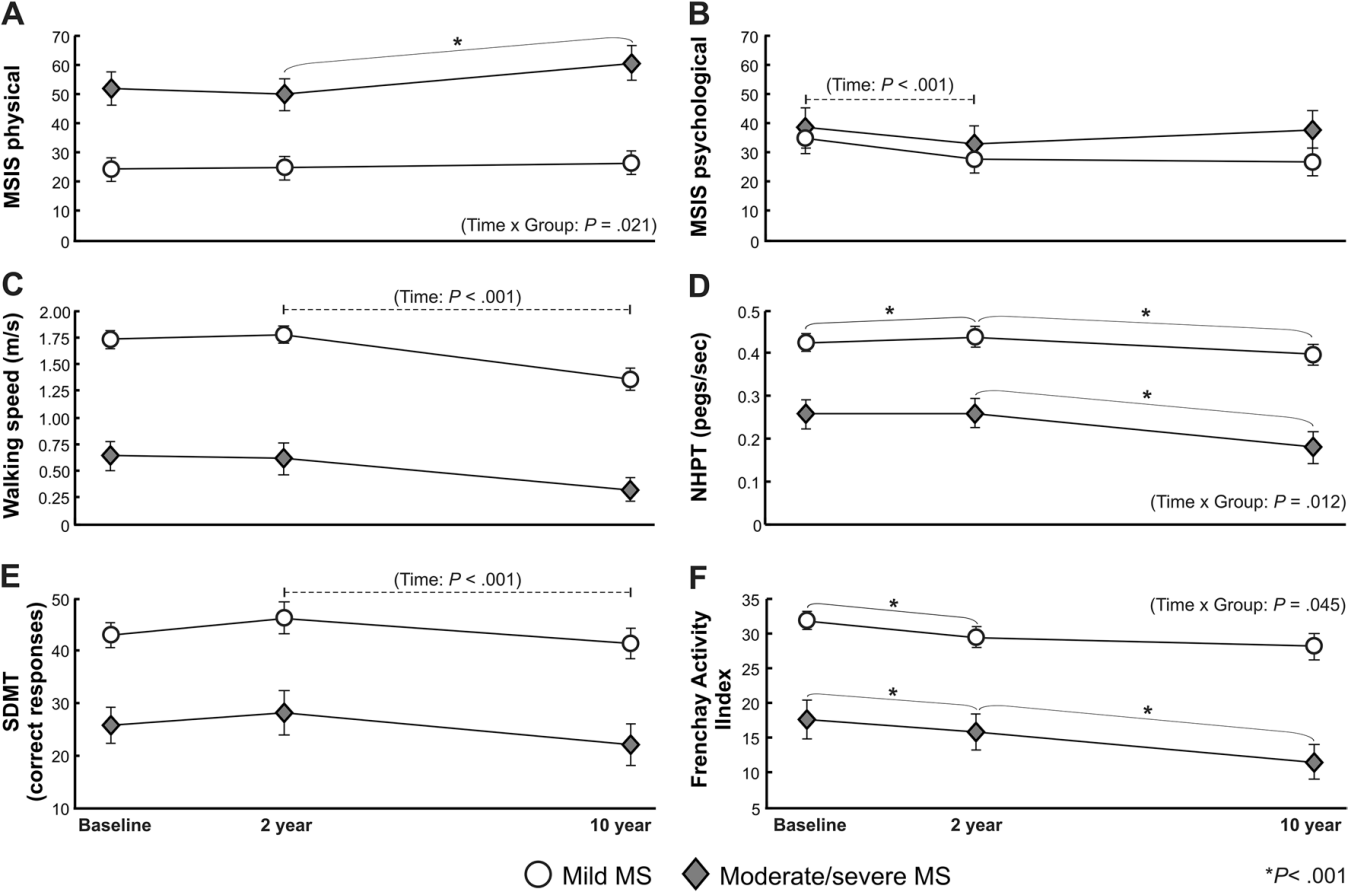
**a** MSIS physical score, **b** MSIS psychological score, **c** walking speed, **d** NHPT, **e** SDMT, and **f** Frenchay Activities Index for individuals with mild and moderate/severe MS at baseline, 2 and 10-year follow-up. Data are plotted as the mean and 95% confidence intervals. *MSIS* The Multiple Sclerosis Impact Scale, *NHPT* Nine-Hole Peg Test, *SDMT* Symbol Digit Modalities Test

The physical impact of MS remained unchanged across the study period in the mild group (*P* > 0.288), whereas the moderate/severe group reported a 22% increase in the physical impact of MS between the 2- and 10-year follow-ups (*P* < 0.001, Fig. 2a, Table 4). The pattern of change of the psychological impact of MS was similar in both groups (time: *P* < 0.001, Fig. 2b, Table 4), revealing a significant reduction between baseline and the 2-year follow-ups (*P* < 0.001), while no change was seen between the 2- and 10-year follow-ups.

**Table 4:** Perceived impact of MS and functioning for individuals with mild and moderate/severe MS at baseline, 2 and 10-year follow-up

	Mild MS (*n* = 101)			Moderate/severe MS (*n* = 54)			*P* value		
	Baseline	2 year	10 year	Baseline	2 year	10 year	Group	Time	Interaction
MSIS physical (0–100)	24.2 (20.0–28.3)	24.6 (20.6–28.6)	26.4 (22.3–30.6)	51.7 (46.1–57.4)	49.7 (44.3–55.1)	60.5 (54.8–66.3)	**<** **0.001**	**<** **0.001**	**0.021**
MSIS psychological (0–100)	34.7 (29.6–39.8)	27.7 (23.1–32.3)	26.7 (22.0–31.3)	38.4 (31.5–45.3)	32.7 (26.4–39.0)	37.7 (31.3–44.1)	0.060	**<** **0.001**	0.178
Timed 25 foot walk (m/s)	1.73 (1.65–1.81)	1.77 (1.69–1.85)	1.36 (1.25–1.46)	0.64 (0.51–0.78)	0.61 (0.47–0.76)	0.32 (0.21–0.44)	**<** **0.001**	**<** **0.001**	0.091
Nine-hole peg test (pegs/sec)	0.42 (0.40–0.44)	0.44 (0.42–0.46)	0.40 (0.37–0.42)	0.26 (0.22–0.29)	0.26 (0.22–0.29)	0.18 (0.14–0.22)	**<** **0.001**	**<** **0.001**	**0.012**
SDMT (correct responses)	42.9 (40.5–45.4)	46.3 (43.2–49.4)	41.4 (38.4–44.3)	25.8 (22.4–29.1)	28.1 (23.9–32.3)	22.1 (18.1–26.1)	**<** **0.001**	**<** **0.001**	0.300
Fatigue Severity Scale (0–7)	4.3 (4.0–4.6)	4.2 (3.9–4.5)	4.4 (4.1–4.8)	5.3 (5.0–5.7)	5.0 (4.6–5.4)	5.0 (4.6–5.5)	**<** **0.001**	0.085	0.126
Frenchay Activities Index (0–45)	31.8 (30.6–33.1)	29.4 (27.9–30.9)	28.1 (26.2–30.0)	17.7 (14.9–20.5)	15.8 (13.3–18.4)	11.5 (9.0–14.0)	**<** **0.001**	**<** **0.001**	**0.045**

Data are presented as mean and 95% confidence interval

*MSIS* Multiple Sclerosis Impact Scale, *MS* Multiple Sclerosis, *SDMT* Symbol Digit Modalities Test

### Changes in walking ability, cognitive processing, manual dexterity, fatigue, and signs of depression

Both groups declined in walking, cognitive processing, and manual dexterity between 2 and 10 years (*P* < 0.001, Fig. 2c–e, Table 4). However, compared to the mild group, the moderate/severe group demonstrated a larger relative decline in walking speed (50 vs. 23%), cognitive processing (21 vs. 11%), and manual dexterity (31 vs. 9%). The level of fatigue appeared unchanged across the study period for both groups (time: *P* = 0.085, Table 4). The proportion experiencing signs of depression was unaltered throughout the study period in moderate/severe MS (baseline: 29%, 2 years: 22%, 10 years: 25%, *P* > 0.167), while fewer in the mild MS group experienced signs of depression at the 10-year follow-up compared to the 2-year follow-up (23 vs. 12%, *P* = 0.021, Table 3).

### Changes in social/lifestyle activities and activities of daily living

While the mild MS group only declined their participation in social/lifestyle activities between baseline and the 2-year follow-up (*P* < 0.001), the moderate/severe group declined gradually across the study period (baseline vs. 2 years: 11%, 2 vs. 10 years: 27%, *P* < 0.001, Fig. 2f, Table 4). For both groups, dependency in personal ADL was unchanged in the short term, but increased significantly between the 2- and 10-year follow-up (*P* < 0.001, Table 3). A short-term increase in instrumental ADL dependency was demonstrated for moderate/severe MS (baseline vs. 2 year: 73 vs. 87%, *P* < 0.001), while the mild MS group increased their dependency in the long term (2 vs. 10 year: 16 vs. 41%, *P* < 0.001).

## Discussion

Our findings shed light on the different courses of disability between the mild and moderate/severe stages of MS. In line with the previous long-term longitudinal studies [3, 5], both severity groups in our study increased their mean EDSS scores with approximately 1–2 points over a decade. However, the consequences of disease progression on overall disability were more prominent in individuals with moderate/severe MS compared to those with mild MS.

Our results show that only the moderate/severe MS group perceived increased physical impact of MS, increased wheel-chair dependency, and reduced participation in social/lifestyle activities across the 10 years. In line with this, the results of performance tests demonstrated a larger decline in walking ability, cognitive processing, and manual dexterity in the moderate/severe group compared to the mild group. These findings contradict the previous results by Wynia et al. [8] who found a more pronounced decline in self-reported disability across 5 years in a mild disease severity group of MS (EDSS 0 to < 4.5) compared to moderate (EDSS ≥ 4.5 to < 7.0) and severe MS (EDSS ≥ 7.0). We believe that the prolonged follow-up in our study and the fact that we included both self-reported and performance-based tests to monitor changes in disability contributed to the detection of increased disability in the moderate/severe group. Our findings highlight the importance of developing a variety of interventions that can be applied across the spectrum of disease severity within the clinical care of MS management. This is particularly important for more severe MS due to the devastating effects of impaired physical [29] and cognitive functioning [30] and their negative impact on disease burden in this subgroup [31]. While there is a large body of research supporting the benefits of exercise for walking impairments in PwMS [32], the optimal treatment strategies for manual dexterity [33] and cognition are still lacking [34]. Therefore, there is an urgent need to develop rehabilitation services to impede the progression of disability in manual dexterity and cognition.

Similar to the previous findings [5], we found increased dependency in personal and instrumental ADL in both groups across the study period. Interestingly, despite increased dependency in ADL between the 2- and 10-year follow-up, the mild MS group sustained their participation in social/lifestyle activities, and 97% remained independent walkers at the 10-year follow-up. It is plausible that adequate compensatory strategies, including personal behavior and modification of environmental factors [35], could have enabled maintenance in social participation in the mild group. To better understand and provide adequate support for PwMS in different stages of MS, we recognize the need for longitudinal and qualitative studies to investigate how psychological factors including coping strategies evolve from the onset of the MS diagnosis and across disease progression.

Despite increased physical disability, psychological impact of MS remained at the same level across 10 years in both severity groups. These results are similar to the previous findings showing a decline in the physical aspects of quality of life in PwMS [4, 6, 7], while psychological scores remained stable [7, 8] or even improved [6]. These results could be derived from adequate use of coping strategies (i.e., problem-solving and seeking social support) in this cohort of PwMS to address the psychological impact of MS [36]. Alternatively, these findings could reflect a response shift in the present cohort, i.e., when individuals experience changes in health (e.g., decline in physical capacity), they may reevaluate their conception of health [37]. Our findings and previous findings [5, 10] show that a large and unchanged proportion of PwMS do experience fatigue and signs of depression over 10 years.

The large proportion of our sample receiving immunomodulatory treatment at baseline needs to be taken into account when interpreting the study results. The increase in EDSS scores in our study was similar to previous 10-year follow-ups monitoring cohorts, where a low proportion received immunomodulatory treatment [3, 5]. The similarities in disease progression between our cohort and previous cohort studies might indicate that changes in severity of MS, as measured with EDSS, occur regardless of immunomodulatory treatment. Although immunomodulatory treatment has shown a promising effect on disease progression its long-term effects on disability in PwMS need to be determined [13].

The strengths of this study were the short- and long-term follow-ups and the use of performance-based tests combined with self-reported evaluation of a broad spectrum of MS disability. The drop-out of 29% in this study was also low with respect to the 10-year follow-up. Nevertheless, some methodology considerations require attention. First, PwMS were consecutively recruited to this study from one MS centre at a university hospital in an urban area, which may limit the generalizability of this work. On the other hand, our sample showed similar age, gender distribution, and work status as reported for PwMS in Sweden in two recent population-based studies [5, 38]. Finally, as those lost to follow-up were older and to a larger extent lived alone with a higher disability level, our results may underestimate the decline in functioning and disability among PwMS.

To conclude, although we observed a decline in functioning irrespective of disease severity over 10 years, only individuals with moderate/severe MS demonstrated a long-term increase in perceived physical impact of MS, increased wheel-chair dependency, and reduced participation in social/lifestyle activities. These findings accentuate the importance of developing a variety of interventions that can be applied across the spectrum of disease severity. Our results also support the previous findings that, despite increased physical disability, the psychological impact of MS, fatigue, and depression remain unaltered over time.
